# The GLIM Criteria Represent a More Appropriate Tool for Nutritional Assessment in Patients With Crohn's Disease

**DOI:** 10.3389/fnut.2022.826028

**Published:** 2022-03-28

**Authors:** Yong Li, Ziheng Peng, Duo Xu, Yu Peng, Xiaowei Liu

**Affiliations:** ^1^Department of Gastroenterology, Xiangya Hospital, Central South University, Changsha, China; ^2^Hunan International Scientific and Technological Cooperation Base of Artificial Intelligence Computer Aided Diagnosis and Treatment for Digestive Disease, Xiangya Hospital, Central South University, Changsha, China; ^3^Research Center for Geriatric Disorder, Xiangya Hospital, Central South University, Changsha, China

**Keywords:** GLIM, NRS 2002, Crohn's disease, nutritional assessment, nutrition therapy

## Abstract

**Background:**

The early recognition of malnutrition is essential for improving the prognosis of patients with Crohn's disease (CD). The Global Leadership Initiative on Malnutrition (GLIM) criteria represent a new consensus for the diagnosis of malnutrition but need to be validated in CD. The aims were to explore the related factors of malnutrition in CD and explore whether GLIM-positive patients who did not meet the Nutritional Risk Screening 2002 (NRS 2002) would benefit from nutritional treatment.

**Methods:**

This study retrospectively enrolled patients with CD at the Gastroenterology Department of Xiangya Hospital Central South University between March 2020 and March 2021. After bioelectrical impedance analysis, all patients underwent nutritional screening and diagnosis using the NRS 2002 and GLIM criteria, respectively. Multivariable analysis was performed to evaluate risk factors related to malnutrition in patients with CD. A multivariable Cox hazard model was used to assess the association between nutritional therapy and prognostic outcomes.

**Results:**

Of the 118 patients included, fifty were classified as having a high malnutrition risk according to the NRS 2002, while 76 were diagnosed with malnutrition by the GLIM criteria. Multivariate analysis showed that a high malnutrition risk was independently associated with the L4 phenotype [odds ratio (OR) (95% confidence interval (CI)) = 4.718 (1.108, 20.10), *p* = 0.036] and Crohn's Disease Activity Index (CDAI) [OR (95% CI) = 1.018 (1.007, 1.029), *p* = 0.002] based on the NRS 2002. The age at onset [OR (95% CI) = 0.828 (0.699, 0.980), *p* = 0.028] and CDAI [OR (95% CI) = 1.111 (1.034, 1.195), *p* = 0.004] were regarded as independent risk factors related to malnutrition, as determined by the GLIM criteria. Among 26 GLIM+/NRS− patients, significantly more patients who received nutritional support achieved 6-week remission than patients who did not (100 vs. 71.4%, *p* < 0.05). The 6-week remission risk in patients treated with nutrition therapy was more than 4-fold higher than those without nutritional therapy.

**Conclusion:**

The GLIM criteria could diagnose more malnourished patients with CD who are not positively screened by the NRS 2002, among whom nutritional support therapy would be beneficial for disease remission. The new criteria should be more appropriate for assessing the nutritional status of patients with CD.

## Introduction

Crohn's disease (CD) is a chronic, relapsing, inflammatory disorder of the digestive tract that can lead to protein loss due to the presence of intestinal leaks. Several studies have reported that 20–40% of outpatients with CD present with specific nutrient deficiencies (1, 2).

Nutritional interventions may improve the outcomes of patients with CD, especially those with severe CD. Screening for and managing malnutrition by an appropriately trained multidisciplinary team is suggested in the European Society for Clinical Nutrition and Metabolism (ESPEN) guidelines (3). Previous researchers have demonstrated increased risks of venous thromboembolism (4), Non-elective surgery (5), longer hospital stays (5), and increased mortality in malnourished patients with CD (6). A meta-analysis revealed that the combination of enteral nutrition with biological agents was 2.4 times more effective in maintaining clinical remission than single treatments (7), suggesting that nutritional therapy plays an important role in the prognosis of patients with CD. However, nutritional treatment is not indicated in every patient, which requires nutritional screening and assessment. Therefore, early nutritional assessment is important in patients with CD.

The Nutritional Risk Screening 2002 (NRS 2002) is recommended by ESPEN guidelines for the nutritional screening in hospitalized patients (8). It classifies the severity of patients' disease in combination with the degree of malnutrition. Raslan et al. (9) assessed three nutritional screening tools for the measurement of nutritional risk, and the NRS 2002 was the best validated. Similarly, several studies have confirmed that the NRS 2002 was suitable for hospitalized Chinese patients (10, 11). Although the NRS 2002 saves time and is easy operation to apply, it is greatly affected by the patient's body mass index (BMI), which results in some malnourished patients with a normal BMI being easily missed. Therefore, there is still a need to find more suitable tools to screen and evaluate the nutritional status of patients with CD.

The Global Leadership Initiative on Malnutrition (GLIM) has been proposed to allow for comparisons and malnutrition diagnoses in regions that use a variety of assessment methods (12). The GLIM is a new diagnostic framework that focuses on building a global consensus around diagnostic criteria for malnutrition in adults (13, 14). Unlike the NRS 2002, the GLIM also assesses the muscle mass of patients. Recent studies have found that the patients with CD with a normal BMI may also suffer from sarcopenia (15). Their nutritional status may be easily overlooked by clinicians. The GLIM has not yet been applied in patients with various diseases, including CD, nor has its predictive value regarding outcomes in these patients. Further research is needed to determine the relevance of these criteria in clinical practice.

This study aims to explore the related factors of malnutrition in CD and to further evaluate whether GLIM-positive patients with CD who are not positively screened by the NRS 2002 can benefit from nutritional treatment.

## Methods

### Patients Selection

This was a retrospective analysis of all adult patients who underwent assessment for CD at Central South University Xiangya Hospital between March 2020 and March 2021. Patients in the study population were newly diagnosed by a multidisciplinary inflammatory bowel disease (IBD) team composed of gastroenterologists, radiologists, pathologists, and dietitians, according to the guidelines established by the European Crohn's and Colitis Organization (ECCO) (16). Eligible subjects aged 18–60 years with a disease duration of 3 months, or longer were screened. No patients had indications for surgical intervention or other concomitant diseases. All eligible patients received infliximab (IFX) via intravenous administration. We excluded patients with anasarca, pregnant women, and those unable to undergo anthropometric measurements for various reasons. In addition, patients who were not treated according to the drug instructions (e.g., dose or frequency of drug administration), who were lost to follow-up or who had incomplete medical data available were excluded.

### Data Collection

Clinical and demographic data were collected from the electronic records within the first 48 h of admission at the patients' bedside by the clinicians. The plasma C-reactive protein (CRP) level was used as a specific measure for the GLIM etilogical criteria of inflammation, considering that all patients had an acute or chronic active disease burden (17). Additional nutritional therapy was collectively decided upon by the dietitians and clinicians, referring to the NRS 2002 results and the patient's decision.

**Figure 1:**
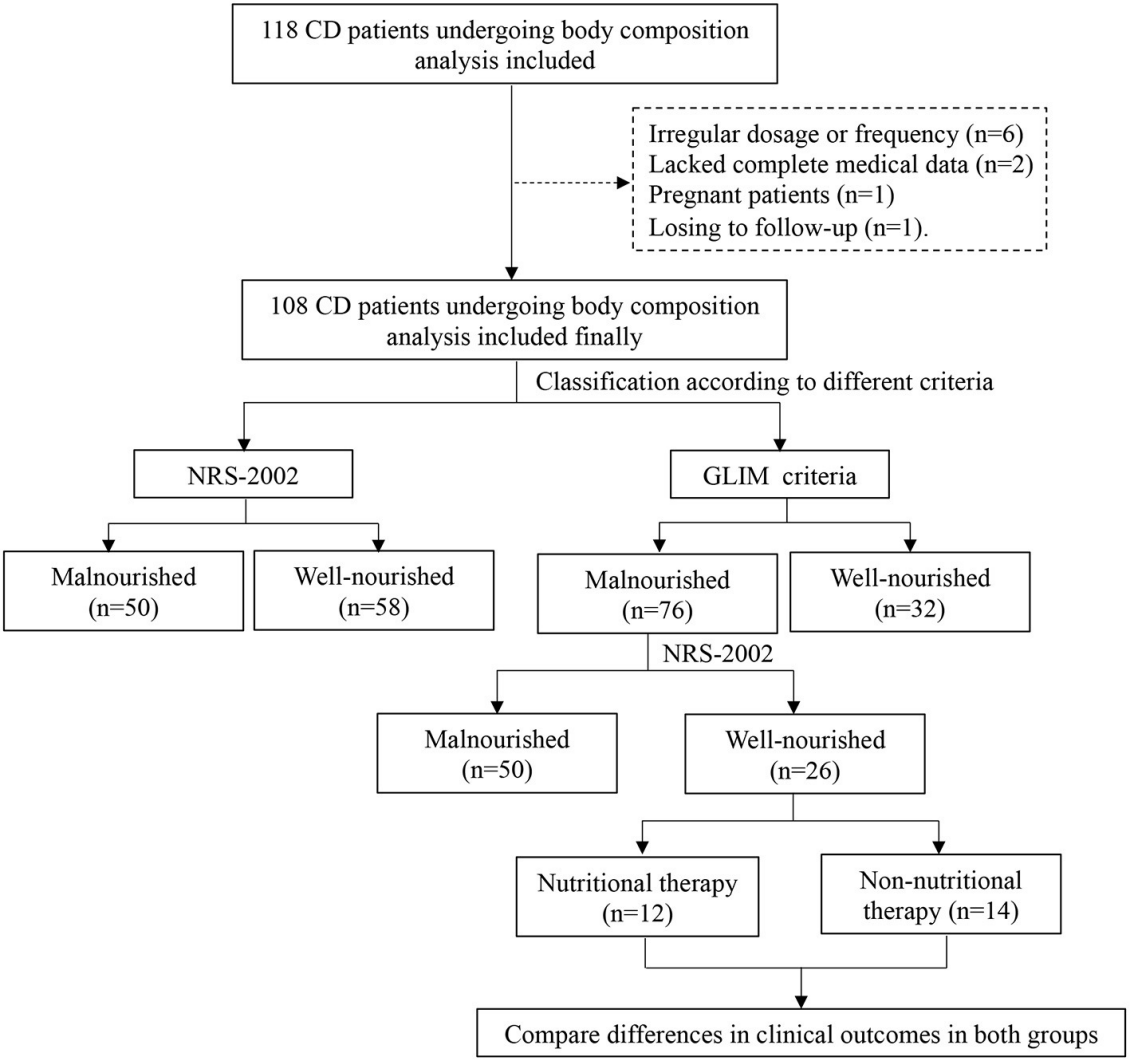
A flow chart of the process of this study.

For the nutritional assessment, trained dietitians evaluated the weight history, diet history, and body composition of the patients. Height and body weight were measured with a scale and stadiometer at the hospital. Eligible patients were required to be barefoot and wear as little clothing as possible (17). The weight loss of the patient within 6 months before hospitalization, was obtained to calculate the percentage of weight loss. A decrease in food and energy intake within 1 week before admission was assessed. A tetrapolar multifrequency bioelectrical impedance analysis (BIA, Tanita, MC-180, Tokyo, Japan), set at a 50-kHz current frequency, was used to assess the body composition after voiding to determine the fat-free mass index (FFMI) (18). Before the measurement of BIA, patients were asked to refrain from performing any exercise, eating, and drinking fluids, including water, for at least 3 h, and were required to empty their bladder in time. During the measurements, patients were barefoot and remained stationary. A flow chart of the study process was shown in the Figure 1.

### Definition

The primary outcome of this study was 6-week remission which was defined as a Crohn's Disease Activity Index (CDAI) <150 at week 6, without the need for any treatment escalation meaning any additional therapy or surgery during this time (19). The occurrence of CD-related surgical treatment and unplanned hospital admission within the first 6 weeks of IFX treatment were considered secondary endpoints.

In our study, if the patient's enteral nutrition intake exceeded 50% within 6 weeks, nutritional therapy was considered (20). By comparing the value of the actual total doses taken divided by the total doses prescribed during the 6-week period, we determined whether the actual intake was more than 50% (21). The actual total doses taken were calculated by the actual purchase records from the outpatient. The dietitians calculated the daily energy intake of each patient according to the ESPEN guidelines (22) and then converted it into the daily dose of enteral nutrition intake, which was the daily prescribed doses of the patient. The total doses prescribed during the 6 weeks were equal to the daily doses multiplied by the time (42 days). The guidelines require that the energy requirements of patients with CD are similar to those of the healthy population, about 25–30 kcal/kg/d (22). And 25 kcal/kg/d were selected in our hospital.

The Montreal classification was applied to define the location and behavior of the CD (23). The location was classified into four categories, including the terminal ileum (L1), colon (L2), ileocolon (L3) and upper gastrointestinal location (L4). The L4 phenotype was defined as involving esophagogastroduodenal, jejunal, or proximal ileal disease anatomically (24). The establishment of the diagnosis and classification was based on the findings of computed tomography enteroclysis, double-balloon enteroscopy and capsule endoscopy, as appropriate (3). The behavior of the disease included nonstricturing and nonpenetrating (B1), stricturing (B2), and penetrating (B3).

The GLIM includes three phenotypical criteria (weight loss, low BMI, and reduced muscle mass) and two etiological criteria (reduced food intake or absorption, and increased disease burden or inflammation). If a patient met at least one phenotypical criterion and one etiological criterion, malnutrition was diagnosed (12). Only one phenotypical criterion for this grade needed to be met to grade a patient's nutritional severity.

The details of the GLIM are as follows (12):

1. Weight loss: A nonvolitional weight loss of 5–10% within the past 6 months, or 10–20% beyond 6 months was defined as moderate malnutrition. A nonvolitional weight loss of >10% within the past 6 months, or >20% prior to the past 6 months was considered to indicate severe malnutrition.
2. BMI: The BMI cut-offs for malnutrition risk were <20 kg/m^2^ if <70 years, and <22 kg/m^2^ if ≥70 years, which was defined as moderate malnutrition. A BMI of <18.5 kg/m^2^ for those aged <70 years, and <20 kg/m^2^ for those aged ≥70 years, was considered to incadite severe malnutrition.
3. Reduced muscle mass: The parameter was assessed by the FFMI in this study. The FFMI cut-offs for malnutrition risk were <17 kg/m^2^ for men and <15 kg/m^2^ for women.
4. Reduced food intake or absorption: That was defined as an intake of 50% or less of energy requirements for >1 week, or any reduction for >2 weeks.
5. Increased disease burden or inflammation: Chronic or recurrent mild-to-moderate inflammation was likely to be associated with malignant disease or any disease that was considerded chronic or recurrent.

The NRS 2002 is based on the patient's nutritional status (including weight loss, BMI, and general condition or food intake) and disease severity (stress metabolism due to the degree of disease) (25). Each section was scored from 0 to 3 points. A total score ≥3 indicated a risk of malnutrition.

The GLIM+/NRS+ group included patients with CD who met the GLIM and NRS 2002, while the GLIM-/NRS− group inclued those who did not fulfill both sets of criteria. The patients who met the GLIM criteria but were negatively identified by the NRS 2002 were included in the GLIM+/NRS− group. And well-nourished patients with a high risk of malnutrition were included in the GLIM-/NRS+ group.

### Statistical Analyses

Continuous variables, expressed as medians and interquartile ranges (IQR), were analyzed using Student's *t* test or nonparametric tests (the Mean-Whitney test) for those with a nonnormal distribution. Categorical variables were expressed as *n* (%). Differences between categorical variables were analyzed using the chi-square test and Fisher's exact probability test. Multivariable logistic regression analysis was performed to evaluate risk factors related to malnutrition in patients with CD. Variables with a *p* value < 0.01 in the univariate analysis were included in the multivariate logistic regression analysis. However, BMI, weight, and FFMI were not considered in the multivariate analysis because they were closely related to the criteria mentioned in this study. A multivariable Cox proportional hazard model was used to assess the association of nutritional therapy with prognostic outcomes. All reported *p* values are two-tailed, and *p* < 0.05 indicated statistical significance. Analyses were performed with SPSS software (version 26.0).

## Results

### Characteristics of the Study Population

There were 118 patients screened for inclusion in this study. Of these, six did not receive treatment according to the instructions, two lacked complete medical data, one patient was pregnant, and one patient was lost to follow-up. Finally, 108 patients with CD who received IFX were enrolled. Of those included, ~69.4% were male, and three-fifths exhibited stenosis. They had a median BMI of 18.3 kg/m^2^ (IQR, 16.6–20.2 kg/m^2^). In addition, 54.6% of patients presented with perianal lesions. The baseline characteristics of the patients were presented in Table 1.

**Table 1:** Baseline characteristic of final cohort.

**Variable**	**Final cohort (*n* = 108)**
Male, No. (%)	75 (69.4%)
Height, median (IQR), cm	168.0 (160.0, 172.3)
Weight, median (IQR), kg	50.4 (45.3, 56.8)
BMI, median (IQR), kg/m^2^	18.3 (16.6, 20.2)
FFMI, median (IQR), kg/m^2^	15.6 (13.9, 16.9)
Age of onset, median (IQR), year	27.0 (17.0, 35.5)
Age at diagnosis, median (IQR), year	31.0 (19.0, 38.3)
Disease course, median (IQR), month	12.0 (3.0, 48.0)
Location, No. (%)	
L1	17 (15.7%)
L2	14 (12.9%)
L3	61 (56.5%)
L4	52 (48.1%)
Behavior, No. (%)	
B1	37 (34.3%)
B2	64 (59.3%)
B3	21(19.4%)
Perianal lesions, No. (%)	59 (54.6%)
Smoker, No. (%)	26 (24.1%)
Surgical history, No. (%)	33 (30.6%)
CDAI score, median (IQR)	188.3 (131.1, 250.6)
Serological examination	
White blood cell, median (IQR), 10^9^/L	6.1 (4.4, 7.4)
NLR, median (IQR)	3.1 (2.2, 4.8)
LMR, median (IQR)	2.5 (1.7, 3.5)
PLR, median (IQR)	242.5 (177.9, 354.2)
Serum albumin, median (IQR), g/L	38.5 (33.8, 41.8)
CRP, median (IQR), mg/L	12.9 (4.2, 34.2)
Fibrinogen, median (IQR), g/L	4.1 (3.0, 4.9)

*BMI, body mass index; FFMI, free fat mass index; CDAI, crohn's disease activity index score; CRP, C-reactive protein; NLR, neutrophil-to-lymphocyte ratio; LMR, lymphocyte-to-monocyte ratio; PLR, platelet-to-lymphocyte ratio; IQR, interquartile range*.

### Analysis of Related Factors of High Malnutrition Risk in Patients With CD

A high malnutrition risk existed in 46.3% of participants at the time of admission according to the NRS 2002 criteria. The patients at a high risk had lower FFMI levels (*p* < 0.05). Notably, these patients had a significant trend toward more involvement of the upper gastrointestinal tract (*p* < 0.05). In addition, the patients at a high risk tended to present with more severe inflammation, which manifested as higher CDAI scores and CRP levels (*p* < 0.05). On serological examinations, those in the high risk group had lower albumin levels, as well as LMR (lymphocyte-to-monocyte ratio) levels, and higher fibrinogen levels (*p* < 0.05).

The multivariate analysis with logistic regression showed that the L4 phenotype [odds ratio (OR) (95% confidence interval (CI)) = 4.718 (1.108, 20.10), *p* = 0.036] and an elevated CDAI [OR (95% CI) = 1.018 (1.007, 1.029), *p* = 0.002] were related factors of a the high malnutrition risk in CD. As presented in Tables 2, 3, both groups were compared based on the NRS 2002.

**Table 2:** Comparison of patients according to the presence of malnutrition risk by NRS 2002.

**Variable**	**NRS + group** **(*n* = 50)**	**NRS−group** **(*n* = 58)**	*P* ^*^
Male, No. (%)	31 (62.0%)	44 (75.9%)	0.119
Height, median (IQR), cm	169.0 (158.0, 173.3)	168.0 (160.3, 170.0)	0.601
Weight, median (IQR), kg	46.0 (41.0, 49.8)	56.5 (53.2, 65.7)	0.000
BMI, median (IQR), kg/m^2^	16.8 (15.5, 17.5)	20.3 (19.1, 22.6)	0.000
FFMI, median (IQR), kg/m^2^	14.0 (13.0, 15.0)	16.8 (16.0, 18.0)	0.000
Age of onset, median (IQR), year	25.0 (17.0, 34.0)	30.0 (20.0, 40.5)	0.230
Age at diagnosis, median (IQR), year	28.0 (19.0, 37.0)	31.0 (20.5, 41.0)	0.389
Disease course, median (IQR), month	12.0 (3.0, 54.0)	12.0 (3.0, 30.0)	0.600
Location, No. (%)			
L1	10 (20.0%)	7 (12.1%)	0.259
L2	9 (18.0%)	5 (8.6%)	0.148
L3	26 (52.0%)	35 (60.3%)	0.383
L4	34 (68.0%)	18 (31.0%)	0.000
Behavior, No. (%)			
B1	18 (36.0%)	19 (32.8%)	0.723
B2	28 (56.0%)	36 (62.1%)	0.522
B3	7 (14.0%)	14 (24.1%)	0.184
Perianal lesions, No. (%)	29 (58.0%)	30 (51.7%)	0.514
Smoker, No. (%)	9 (18.0%)	17 (29.3%)	0.170
Surgical history, No. (%)	21(42.0%)	12 (20.7%)	0.017
CDAI score, median (IQR)	204.9 (165.0, 280.6)	154.0 (101.3, 192.0)	0.001
Serological examination			
White blood cell, median (IQR), 10^9^/L	5.7 (4.4, 7.7)	6.3 (4.6, 7.3)	0.673
NLR, median (IQR)	3.4 (2.3, 4.9)	3.0 (2.0, 4.8)	0.455
LMR, median (IQR)	2.3 (1.6, 2.8)	2.8 (2.0, 4.3)	0.028
PLR, median (IQR)	262.5 (201.8, 373.3)	235.3 (136.2, 336.4)	0.246
Serum albumin, median (IQR), g/L	35.1 (31.7, 40.8)	39.9 (36.2, 43.7)	0.003
CRP, median (IQR), mg/L	19.0 (10.7, 70.8)	6.6 (3.4, 16.6)	0.002
Fibrinogen, median (IQR), g/L	4.5 (3.3, 5.4)	3.7 (2.6, 4.5)	0.034

*NRS, Nutritional risk screening 2002; BMI, body mass index; FFMI, free fat mass index; CDAI, crohn's disease activity index score; CRP, C-reactive protein; NLR, neutrophil-to-lymphocyte ratio; LMR, lymphocyte-to-monocyte ratio; PLR, platelet-to-lymphocyte ratio; IQR, interquartile range*.

* *Mann-Whitney U test for continuous variables and chi-square for proportions*.

**Table 3:** Multivariate analysis of factors related to high malnutrition risk by NRS 2002 and malnutrition by GLIM in patients with CD.

**Criterion**	**Variable**	**Multivariate analysis**	
		**OR (95% CI)**	***p***
NRS 2002	L4	4.718 (1.108, 20.10)	0.036
	CDAI score	1.018 (1.007, 1.029)	0.002
GLIM	Age of onset	0.828 (0.699, 0.980)	0.028
	CDAI score	1.111 (1.034, 1.195)	0.004

*NRS 2002, Nutritional risk screening 2002; GLIM, global leadership initiative on malnutrition; CDAI, crohn's disease activity index score; OR, odds ratio; CI, confidence intervals*.

### Analysis of Related Factors of Malnutrition in Patients With CD

The study population was reclassified according to the GLIM criteria. In terms of weight, BMI, and FFMI (*p* < 0.05), the results were similar to those using the NRS 2002 as the classification criterion. Besides, malnourished patients were relatively younger at onset age than the well-nourished patients and exhibited higher CDAI and CRP levels (*p* < 0.05). No differences were found in other serological tests. In contrast, the age at onset [OR (95% CI) = 0.828 (0.699, 0.980), *p* = 0.028] and the CDAI score [OR (95% CI) = 1.111 (1.034, 1.195), *p* = 0.004] were regarded as the independent risk factors related to the nutritional status of patients according to the GLIM criteria. The detailed data were provided in Tables 3, 4.

**Table 4:** Comparison of patients according to the presence of malnutrition by GLIM criteria.

**Variable**	**GLIM+ group (*n* = 76)**	**GLIM−group** **(*n* = 32)**	*P* ^*^
Male, No. (%)	50 (65.8%)	25 (78.1%)	0.204
Height, median (IQR), cm	169.0 (159.4, 173.0)	166.5 (160, 170)	0.465
Weight, median (IQR), kg	47.5 (44.1, 52.9)	58.8 (53.7, 66.4)	0.000
BMI, median (IQR), kg/m2	17.3 (15.8, 18.5)	21.5 (19.9, 23.7)	0.000
FFMI, median (IQR), kg/m2	14.5 (13.6, 16.0)	17.5 (17.0, 18.3)	0.000
Age of onset, median (IQR), year	25.0 (17.0, 31.8)	33.5 (24.8, 46.3)	0.017
Age at diagnosis, median (IQR), year	27.5 (19.5, 36.5)	35.0 (25.8, 47.0)	0.052
Disease course, median (IQR), month	12.0 (3.3, 57.0)	6.5 (1.8, 27.0)	0.198
Location, No. (%)			
L1	12 (15.8%)	5 (15.6%)	0.983
L2	13 (17.1%)	1 (3.1%)	0.097
L3	41 (53.9%)	20 (62.5%)	0.413
L4	40 (52.6%)	12 (37.5%)	0.151
Behavior, No. (%)			
B1	29 (38.2%)	8 (25.0%)	0.670
B2	44 (57.9%)	20 (62.5%)	0.656
B3	14 (18.4%)	7 (21.9%)	0.679
Perianal lesions, No. (%)	45 (59.2%)	14 (43.8%)	0.141
Smoker, No. (%)	14 (18.4%)	12 (37.5%)	0.061
Surgical history, No. (%)	24 (31.6%)	9 (28.1%)	0.722
CDAI score, median (IQR)	197.6 (152.4, 270.9)	141.7 (99.7, 198.0)	0.029
Serological examination			
White blood cell, median (IQR), 10^9^/L	6.1 (4.4, 7.9)	5.9 (4.6, 7.0)	0.711
NLR, median (IQR)	3.2 (2.2, 4.9)	3.0 (1.9, 5.0)	0.640
LMR, median (IQR)	2.5 (1.7, 2.9)	2.8 (1.7, 4.6)	0.235
PLR, median (IQR)	245.6 (181.6, 340.5)	219.8 (133.4, 376.7)	0.784
Serum albumin, median (IQR), g/L	37.7 (33.4, 41.6)	38.9 (35.9, 43.6)	0.439
CRP, median (IQR), mg/L	17.2 (6.6, 47.8)	6.0 (3.6, 9.2)	0.005
Fibrinogen, median (IQR), g/L	4.4 (3.1, 5.2)	3.7 (2.7, 4.2)	0.063

*GLIM, Global leadership initiative on malnutrition; BMI, body mass index; FFMI, free fat mass index; CDAI, crohn's disease activity index score; CRP, C-reactive protein; NLR, neutrophil-to-lymphocyte ratio; LMR, lymphocyte-to-monocyte ratio; PLR, platelet-to-lymphocyte ratio; IQR, interquartile range*.

* *Mann-Whitney U test for continuous variables and chi-square for proportions*.

We found that ~81.6% of the enrolled patients had severe malnutrition according to the GLIM criteria. The L4 phenotype was more common among patients with severe malnutrition than those with moderate dystrophy (*p* = 0.031). Similarly, they also had relatively higher CDAI scores and CRP levels (*p* < 0.05). The data were presented in the Supplementary Table 1.

### Effect of Nutritional Therapy on Clinical Outcomes

In the study, patients were classified depending on whether they fulfilled the GLIM and NRS 2002 criteria. The results of classification were presented in Table 5. There were 50 patients with CD who were classified as having malnutrition with a high nutritional risk (i.e., GLIM+/NRS+ group), and 92% of them had received nutritional therapy. Thirty-two were well-nourished patients with a low nutritional risk (i.e., GLIM−/NRS− group), of whom only 9.4% received nutritional therapy. No well-nourished patients with a high nutritional risk (i.e., GLIM-/NRS+ group) were identified.

**Table 5:** Results of nutritional intervention in different groups of patients with CD.

**Variable**	**Nutritional intervention**	
	**Yes**	**No**
GLIM+/NRS+ group	46 (92.0%)	4 (8.0%)
GLIM+/NRS− group	12 (46.2%)	14 (53.8%)
GLIM−/NRS− group	3 (9.4%)	29 (90.6%)
GLIM−/NRS+ group	0 (0.0%)	0 (0.0%)

*GLIM, Global leadership initiative on malnutrition; NRS, nutritional risk screening 2002*.

Additionally, of the 76 patients who met the GLIM criteria, twenty-six were negatively identified by the NRS 2002. These 26 patients were inclueded in the GLIM+/NRS− group. Of these patients, 46.2% (12/26) received nutritional therapy, while the remainder did not. Table 6 showed the baseline data of the patients with CD treated with or without nutritional support. The clinical outcomes in these two groups were compared to further explore whether the patients missed by the NRS 2002 benefited from nutritional therapy. Among the GLIM+/NRS− patients, significantly more patients who received nutritional support achieved remission at 6-weeks than those who did not (100 vs. 71.4%, *p* < 0.05). The 6-week remission risk in patients with CD receiving nutritional therapy was more than 4-fold higher than in those without nutritional therapy after adjustment for age, gender, and disease activity [unadjusted hazard ratio (HR) (95% CI) = 2.610 (1.108, 6.147), *p* = 0.028]; adjusted HR (95% CI) = 4.251 (1.496, 12.08), *p* = 0.007). However, the rates of surgery and unplanned hospitalizations did not differ between the groups.

**Table 6:** Comparison of GLIM+/NRS− patients with or without nutritional therapy.

**Variable**	**GLIM+/NRS−group (*n* = 26)**	**Nutritional therapy (*n* = 12)**	**Non-nutritional therapy (*n* = 14)**	*P* ^*^
Male, No. (%)	18 (69.2%)	8 (66.7%)	10 (71.4%)	0.793
Height, median (IQR), cm	169.5(161.4, 174.3)	168.7 (162.4, 173.1)	169.9 (163.5, 172.8)	0.568
Weight, median (IQR), kg	47.5 (44.1, 52.9)	48.4 (45.2, 54.1)	47.4 (44.9, 53.6)	0.827
BMI, median (IQR), kg/m^2^	17.3 (16.1, 18.1)	17.0 (16.4, 18.0)	16.8 (16.2, 17.9)	0.791
FFMI, median (IQR), kg/m^2^	15.2 (14.4, 16.3)	15.1 (14.4, 16.2)	14.9 (14.5, 16.4)	0.773
Age of onset, median (IQR), year	26.0 (19.0, 32.5)	25.5 (19.0, 33.0)	26.0 (19.5, 33.5)	0.732
Age at diagnosis, median (IQR), year	28.0 (23.0, 38.5)	28.5 (23.0, 37.0)	28.0 (22.5, 38.5)	0.757
Disease course, median (IQR), month	12.0 (3.9, 48.5)	11.0 (4.5, 47.5)	12.2 (3.8, 48.0)	0.636
Location, No. (%)				
L1	4 (15.4%)	2 (16.7%)	2 (14.3%)	0.867
L2	5 (19.2%)	2 (16.7%)	3 (21.4%)	0.759
L3	14 (53.8%)	6 (50.0%)	8 (57.1%)	0.716
L4	12 (46.2%)	6 (50.0%)	6 (42.9%)	0.716
Behavior, No. (%)				
B1	10 (38.5%)	5 (41.7%)	5 (35.7%)	0.756
B2	14 (53.8%)	6 (50.0%)	8 (57.1%)	0.716
B3	5 (19.2%)	2 (16.7%)	3 (21.4%)	0.759
Perianal lesions, No. (%)	15 (57.7%)	7 (58.3%)	8 (57.1%)	0.951
Smoker, No. (%)	5 (19.2%)	2 (16.7%)	3 (21.4%)	0.759
Surgical history, No. (%)	8 (30.8%)	4 (33.3%)	4 (28.6%)	0.793
CDAI score, median (IQR)	198.1 (161.5, 264.8)	197.6 (165.7, 273.3)	200.1 (160.9, 252.6)	0.867
Endpoints				
6-week remission, No. (%)	21 (80.8%)	12 (100.0%)	10 (71.4%)	0.044
Surgery, No. (%)	0 (0.0%)	0 (0.0%)	0 (0.0%)	1.000
Unplanned hospitalization, No. (%)	1 (3.8%)	0 (0.0%)	1 (7.1%)	1.000
Serological examination				
White blood cell, median (IQR), 10^9^/L	5.8 (4.1, 7.5)	6.1 (4.4, 7.3)	5.9 (4.1, 7.8)	0.986
NLR, median (IQR)	3.0 (2.1, 4.5)	3.1 (2.0, 4.3)	3.0 (2.0, 4.2)	0.849
LMR, median (IQR)	2.4 (1.5, 3.1)	2.2 (1.6, 3.1)	2.4 (1.4, 3.0)	0.109
PLR, median (IQR)	225.3 (185.7, 329.4)	231.4 (187.3, 316.4)	221.9 (191.1, 321.7)	0.639
Serum albumin, median (IQR), g/L	36.4 (32.5, 40.7)	36.8 (32.5, 41.3)	36.3 (34.1, 41.0)	0.094
CRP, median (IQR), mg/L	17.3 (6.4, 46.3)	16.9 (6.1, 45.2)	17.6 (6.5, 44.9)	0.783
Fibrinogen, median (IQR), g/L	4.2 (3.2, 5.4)	4.1 (3.2, 5.1)	4.3 (3.1, 5.7)	0.815

*GLIM, Global leadership initiative on malnutrition; BMI, body mass index; FFMI, free fat mass index; CDAI, crohn's disease activity index score; CRP, C-reactive protein; NLR, neutrophil-to-lymphocyte ratio; LMR, lymphocyte-to-monocyte ratio; PLR, platelet-to-lymphocyte ratio; IQR, interquartile range*.

* *Mann-Whitney U test for continuous variables and chi-square for proportions*.

## Discussion

Malnutrition has been shown to affect the prognosis of patients with CD. Thus, it is important to have accurate criteria for the diagnosis of malnutrition in patients with CD. The current study demonstrated that disease phenotype, age of onset, and disease activity were associated with nutritional status in patients with CD treated with IFX. Furthermore, among GLIM+/NRS− patients, nutritional intervention could increase the likelihood of 6-week remission. The choice of the GLIM criteria appeared to be preferable in terms of clinical decision-making regarding nutritional therapy in patients with CD.

In this cohort, 70.4% of the patients with CD were diagnosed with malnutrition according to the GLIM criteria. This proportion appeared to be higher than those reported in recent studies, resulting in a prevalence of malnutrition ranging from 20 to 40% (26–28). This result may have been due to the lack of a consensus on the exact criteria for defining malnutrition in CD, which led to inconsistent and incomparable results. Furthermore, our cases were affected by active disease requiring biological treatment to reduce the offset, thus explaining the high proportion of patients with malnutrition.

This study showed that disease activity was associated with malnutrition regardless of whether the patients with CD were screened or diagnosed by the NRS 2002 or the GLIM criteria, respectively. Indeed, a severe inflammatory state not only disturbs intestinal barrier function, thereby increasing protein loss (29), but also contributes to the promotion of lipid oxidation and thermogenesis induced by the diet, which may lead to a difference in the basal metabolic rate between patients with both active and remissive CD, as shown in previous studies (30, 31).

Beyond that, we identified the L4 phenotype as an important factor of a high malnutrition risk when classified by the NRS 2002. The L4 phenotype was also observed more frequently in patients with severe malnutrition when classified according to the GLIM. Several studies have suggested that patients with the L4 phenotype have a worse prognosis than those with other phenotypes, which manifests as an increased risk for complications, surgery, and further hospitalization (24, 32–34). The small intestine is essential for the digestion and absorption of macro- and micronutrients (35). Intestinal cells impairment hampers the absorption of nutrients in the body (36, 37), which is sufficient to explain the predisposition of L4-phenotype patients to malnutrition.

Surprisingly, our findings also suggested that the age of onset similarly affected the nutritional status of patients. Previous literature have confirmed that younger patients predominantly have upper gastrointestinal involvement (38–40), and more frequently showed in progression to complicated disease states. Our study revealed a high cumulative proportion of patients with the L4 phenotype in the GLIM+ group, but the difference was not statistically significant. This result indicates that the classification of patients aged 18–60 years into age subgroups for further analysis might lead to more novel findings in subsequent studies if the sample size is sufficient. On the other hand, Xiao et al. (41) discovered that muscle mass was positively associated with age in adulthood and started to decrease from the fifth decade. Assessment of muscle mass is a routine item in the GLIM, which might increase the likelihood of young patients being assessed as positive according to that criterion. The studies mentioned above support the credibility of our results.

In this study, the patients were divided into four groups using two sets of criteria to explore which patient population would benefit from nutritional intervention. Our study found mostly concordance in the decisions made by clinicians when the results of the two criteria were concordant. Additionally, no patients were assigned to the GLIM-/NRS+ group. This may be because the GLIM specifically assesses muscle mass in addition to most of the criteria in the NRS 2002. Therefore, the absence of such patients is justified.

However, nutritional therapy for GLIM+/NRS− patients is controversial. If different criteria are applied for clinical decision-making, clinicians will make different choices. Interestingly, our finding further indicated that nutritional therapy was effective in increasing the clinical remission rate among GLIM+/NRS− patients. Dietary therapy with exclusive enteral nutrition has been recommended as the first-line treatment for pediatric patients with CD (42), but the results of studies in adults have diverged. A recent review involing adults with CD evidenced that no differences in the ability of exclusive enteral nutrition and corticosteroids to induce remission (43). Consensus clinical guidelines in Japan recommend routine nutritional treatment for routine use (44). However, excessive nutritional therapy places an additional financial burden on patients and may not have a significant effect. Therefore, elucidating the appropriate indications for nutritional therapy is particularly critical. Previous evidence suggested that nutritional support improved the clinical outcomes of patients with NRS2002 scores higher than 3 (45). Our study considered that patients who were overlooked by the NRS 2002 would also benefit from nutritional therapy, further illustrating that the GLIM criteria may be more suitable in patients with CD. Additionally, the GLIM criteria consider muscle mass, which has been overlooked by the majority of criteria for evaluating malnutrition. A systematic review revealed that up to 60% of IBD patients exhibited skeletal muscle mass depletion (15), which correlated with the blockage of protein synthesis and absorption in patients (46). Interestingly, Adams et al. (47) reported that more than 40% of patients affected by sarcopenia presented with a normal BMI, and that up to 20% were overweight or obese, which were not identified as undernourished by traditional measures. Sarcopenia has been considered to be a meaningful marker of an adverse prognosis in patients with CD (48–50). Recent studies reported that moderate endurance and muscle training were beneficial for patients with quiescent or mildly active CD (51, 52), which may imply the potential impact of improved muscle status on disease activity to some extent. This potential effect is somewhat related to the assessment of muscle mass by the GLIM criteria. Therefore, screening for sarcopenia needs to be highlighted in the nutritional assessment of all patients with CD. The GLIM criteria allow the more timely screening of such potential patients than other tools.

Some limitations should be considered. Since this was a single-center study with a small sample size, subgroup analysis was restricted. Moreover, self-reporting rather than an in-depth historical diet assessment or a rigorous recording process was used to assess food intake. Our study has a pragmatic design that reflects the reality in most clinical practices. In addition, the endpoints chosen for this study were short, and whether nutritional therapy can improve long-term outcomes in these patients requires continued reseach. The cost-benefit ratio of nutritional therapy in these patients will also be another focus of the follow-up studies.

In conclusion, an attempt was undertaken to evaluate the utilization of a new nutritional diagnostic framework in CD. The GLIM criteria could diagnose more malnourished patients with CD who are not screened by the NRS 2002, among whom nutritional support therapy would be beneficial for disease remission. Further prospective cohort studies are warranted to improve the better application of the GLIM for clinical guidance.

## Data Availability Statement

The raw data supporting the conclusions of this article will be made available by the authors, without undue reservation.

## Ethics Statement

The studies involving human participants were reviewed and approved by the Xiangya Hospital of Central South University Ethics Committees, and each subject provided written, informed consent prior to study participation. The patients/participants provided their written informed consent to participate in this study. Written informed consent was obtained from the individual(s) for the publication of any potentially identifiable images or data included in this article.

## Author Contributions

YL, ZP, DX, YP, and XL: study concept and design. ZP and DX: acquisition of data. YL, YP, and XL: analysis and interpretation of data. YL, ZP, and YP: statistical analysis. YL, YP, and XL: drafting of the manuscript. YL, ZP, DX, YP, and XL: critical revision of the manuscript for important intellectual content. All authors approved the final version of the manuscript and including the authorship list.

## Funding

This work was supported by the National Natural Science Foundation of China (Grant No. 81770584).

## Conflict of Interest

The authors declare that the research was conducted in the absence of any commercial or financial relationships that could be construed as a potential conflict of interest.

## Publisher's Note

All claims expressed in this article are solely those of the authors and do not necessarily represent those of their affiliated organizations, or those of the publisher, the editors and the reviewers. Any product that may be evaluated in this article, or claim that may be made by its manufacturer, is not guaranteed or endorsed by the publisher.
