# Supplementary material for: The GLIM Criteria Represent a More Appropriate Tool for Nutritional Assessment in Patients With Crohn's Disease
Source: Front Nutr. 2022 Mar 28;9:826028. doi: 10.3389/fnut.2022.826028 (PMC9000965; doi:10.3389/fnut.2022.826028)
Supplement: Supplementary file 1 [file Table_1.docx]

Supplementary Table 1. **Comparison of patients with different degrees of malnutrition according to the GLIM criteria**

| Variable | Severe malnutrition  group (n=62) | Moderate malnutrition group (n=14) | *P^*^* |
| --- | --- | --- | --- |
| Male, No. (%) | 40 (64.5%) | 10 (71.4%) | 0.622 |
| Height, median (IQR), cm | 169.0 (158.6, 173.9) | 168.0 (164.0, 170.0) | 0.640 |
| Weight, median (IQR), kg | 46.6 (42.0, 50.1) | 55.0 (53.3, 56.1) | 0.001 |
| BMI, median (IQR), kg/m2 | 16.9 (15.5, 17.7) | 19.5 (18.9, 20.9) | 0.000 |
| FFMI, median (IQR), kg/m2 | 14.3 (13.2, 15.6) | 16.2 (15.7, 16.7) | 0.004 |
| Age of onset, median (IQR), year | 23.5 (17.0, 34.0) | 25.0 (15.0, 27.0) | 0.263 |
| Age at diagnosis, median (IQR), year | 27.5 (18.0, 37.0) | 27.0 (15.0, 28.0) | 0.277 |
| Disease course, median (IQR), month | 12.0 (3.0, 57.0) | 12.0 (7.0, 60.0) | 0.735 |
| Location, No. (%) |  |  |  |
| L1 | 10 (16.1%) | 2 (14.3%) | 0.864 |
| L2 | 10 (16.1%) | 3 (21.4%) | 0.634 |
| L3 | 33 (53.2%) | 8 (57.1%) | 0.791 |
| L4 | 29 (48.4%) | 11 (71.4%) | 0.031 |
| Behavior, No. (%) |  |  |  |
| B1 | 22 (35.5%) | 7 (50.0%) | 0.313 |
| B2 | 36 (58.1%) | 8 (57.1%) | 0.950 |
| B3 | 10 (16.1%) | 4 (28.5%) | 0.278 |
| Perianal lesions, No. (%) | 36 (58.1%) | 9 (64.2%) | 0.669 |
| Smoker, No. (%) | 12 (19.4%) | 2 (14.3%) | 0.659 |
| Surgical history, No. (%) | 21 (33.9%) | 3 (21.4%) | 0.366 |
| CDAI score, median (IQR) | 204.9 (161.1, 279.7) | 150.8 (144.8, 157.6) | 0.019 |
| Serological examination |  |  |  |
| White blood cell, median (IQR), 10^9^/L | 5.9 (4.5, 7.7) | 4.7 (3.5, 8.3) | 0.552 |
| NLR, median (IQR) | 3.2 (2.3, 4.8) | 4.8 (2.0, 6.1) | 0.697 |
| LMR, median (IQR) | 2.3 (1.6, 2.8) | 2.8 (2.0, 3.5) | 0.331 |
| PLR, median (IQR) | 258.3 (196.9, 361.2) | 246.2 (240.0, 401.8) | 0.647 |
| Serum albumin, median (IQR), g/L | 35.9 (32.6, 41.4) | 39.6 (36.0, 43.5) | 0.211 |
| CRP, median (IQR), mg/L | 17.8 (9.7, 59.6) | 4.2 (2.1, 4.5) | 0.001 |
| Fibrinogen, median (IQR), g/L | 4.4 (3.2, 5.3) | 11.8 (1.7, 20.5) | 0.577 |
| Abbreviations: GLIM, Global Leadership Initiative on Malnutrition; BMI, body mass index; FFMI, free fat mass index; CDAI, Crohn's Disease Activity Index score; CRP, C-reactive protein; NLR, neutrophil-to-lymphocyte ratio; LMR, lymphocyte-to-monocyte ratio; PLR, platelet-to-lymphocyte ratio; IQR, interquartile range  *Mann-Whitney U test for continuous variables and chi-square for proportions. | | | |
